# Role of Positron Emission Tomography (PET) in the Diagnosis of Musculoskeletal Disorders

**DOI:** 10.3390/jcm14093080

**Published:** 2025-04-29

**Authors:** Raju Vaishya, Jena Amarnath, Prerana Rana, Rajesh Botchu, Abhishek Vaish

**Affiliations:** 1 Department of Orthopaedics and Joint Replacement Surgery, Indraprastha Apollo Hospitals, New Delhi 110076, India; 2 Department of PET Imaging, Indraprastha Apollo Hospitals, New Delhi 110076, India; dramarnath_j@apollohospitals.com (J.A.); prerana@hod.care (P.R.); 3 Department of Musculoskeletal Radiology, Royal Orthopedic Hospital, Birmingham B31 2A, UK; rajesh.botchu@nhs.net; 4 Department of Orthopaedic and Joint Replacement Surgery, Indraprastha Apollo Hospitals, New Delhi 110076, India; drabhishek_vaish@apollohospitals.com

**Keywords:** musculoskeletal disorders, positron emission tomography, radiotracers, [^18^F]-FDG, [^18^F]-NaF, bone metabolism

## Abstract

Musculoskeletal disorders (MSDs) represent a broad spectrum of diseases and injuries that significantly affect the musculoskeletal system and impose a considerable burden on global public health. This review focuses on the landscape of MSD diagnoses and emphasizes the high prevalence of these conditions. Additionally, it recognizes the inadequacies of conventional evaluation methods, including radiography and subjective assessments, when addressing their complex pathophysiology. It also attempts to highlight the promise of positron emission tomography (PET), which offers quantitative insights into metabolic and molecular activities before structural changes become evident. The review focuses on key radiotracers, specifically, fluorodeoxyglucose ([^18^F]-FDG) and sodium fluoride ([^18^F]-NaF), discussing their efficacy in assessing inflammatory processes and bone metabolism. By exploring the abilities of these advanced imaging modalities, we aim to identify the potential of using PET in the early detection and more accurate assessment of MSDs. Furthermore, we provide a brief outline of directions for future research, advocating for the development of novel radiotracers, the integration of multiple imaging modalities, and the application of artificial intelligence in imaging analysis. This review contributes to a deeper understanding of MSDs and underscores the urgent need for innovative diagnostic strategies to improve patient care and outcomes in musculoskeletal health.

## 1. Introduction

Musculoskeletal disorders (MSDs) encompass various diseases and injuries affecting the joints, bones, muscles, nerves, tendons, ligaments, supporting soft tissues, cartilage, and spinal discs, with over 150 diagnoses recognized. Among the most commonly encountered conditions are various forms of arthritis, such as rheumatoid arthritis (RA), osteoarthritis (OA), osteoporosis, osteopenia, sarcopenia, infections, neoplasms, and chronic neck and lower back pain. The prevalence of MSDs places a substantial burden on public health worldwide, ranking as a leading cause of disability, second only to mental health and substance use disorders. Their impact extends across all age groups and manifests through high societal costs, including limited mobility and functional ability, persistent pain, early retirement, reduced societal participation, mental health challenges such as depression, and increased vulnerability to other chronic health conditions.

The current clinical evaluation methods for MSDs primarily rely on physical examinations, serum biomarkers, and subjective pain- and health-assessment questionnaires. While imaging modalities such as radiography, computed tomography (CT), and magnetic resonance imaging (MRI) have traditionally been used, their correlation with clinical signs and treatment responses is weak. This presents a fundamental knowledge gap: precise anatomical imaging alone may not adequately capture the underlying molecular and pathophysiologic processes associated with MSDs. Advanced imaging techniques are critical to providing insights into these disorders’ molecular aspects as they evolve [1].

Positron emission tomography (PET) imaging offers a unique advantage in this context, as it enables quantitative assessment of metabolic and molecular activity, often preceding structural and biochemical changes. To fully leverage the capabilities of PET imaging, higher-resolution anatomical data is essential. Although molecular imaging with PET/CT and PET/MRI has shown promise for evaluating MSDs, studies in this area remain limited.

Given the existing limitations of conventional imaging, and the pressing need for the early and accurate detection of MSDs, it is imperative to explore the integration of advanced imaging modalities such as PET. This review aims to examine the current landscapes of MSD diagnoses, investigate the potential of PET and molecular imaging techniques in enhancing the early diagnosis and monitoring of MSDs, provide recommendations for future research directions to bridge the knowledge gaps in the field of musculoskeletal (MSK) imaging, and improve patient outcomes. By addressing these aims, this review seeks to contribute to a deeper understanding of MSDs as complex “organ unit” i.e., a collection of tissue joined in a structural unit to serve a common function, and the development of more effective diagnostic strategies [2].

## 2. Radiotracers for Musculoskeletal Imaging

A diverse array of PET radiotracers has been developed for MSK imaging, with two particularly notable tracers frequently utilized in clinical practice: fluorodeoxyglucose ([^18^F]-FDG) and sodium fluoride ([^18^F]-NaF).

(A) [^18^F]-Flurodeoxyglucose (FDG)

[^18^F]-FDG is the most commonly employed PET radiotracer. As an analogue of glucose, [^18^F]-FDG provides valuable insights into tissues characterized by elevated glucose uptake and metabolic activity. This property makes [^18^F]-FDG-PET particularly effective in identifying areas of increased metabolic demand that are often associated with inflammatory processes. Activated inflammatory cells exhibit heightened glucose utilization, rendering [^18^F]-FDG a powerful tool for detecting musculoskeletal inflammation and infection. Its application extends to musculoskeletal disorders, including arthritis and other inflammatory conditions.

(B) [^18^F]-Sodium Fluoride (NaF)

On the other hand, [^18^F]-NaF serves as a well-known PET radiotracer that specifically targets bone metabolism. When introduced into the body, the [^18^F]-fluoride ion exchanges with hydroxyl groups in hydroxyapatite crystals present on the surface of the bone matrix, leading to the formation of fluorapatite. The uptake of [^18^F]-NaF thus acts as an effective marker of bone metabolic activity, with a notable concentration in areas of newly mineralizing bone. This characteristic makes it particularly useful in scenarios where [^18^F]-FDG is less effective, such as in certain malignancies—including thyroid and renal cell cancers—where bone metastases are assessed.

Additionally, [^18^F]-NaF offers significant potential for evaluating bone turnover and repair mechanisms in non-oncologic musculoskeletal conditions. It has shown promise in fracture healing, arthritic diseases, and metabolic bone disorders, providing clinicians with critical information regarding bone health and recovery.

The utilization of these radiotracers highlights the growing importance of advanced molecular imaging techniques in understanding and managing musculoskeletal disorders, enabling more precise diagnosis and treatment strategies [2,3,4,5,6]. A summary of the key features of various radiotracers used for MSK imaging is presented in Table 1.

## 3. Imaging Techniques in Various Musculoskeletal Conditions (Figure 1)

### 3.1. Osteoporosis

Osteoporosis, classified by the World Health Organization (WHO) as a skeletal disease characterized by low bone mass and micro-architectural deterioration, significantly increases the risk of fractures. Early diagnosis is essential for effective intervention, and traditional methods such as dual-energy X-ray absorptiometry (DXA) serve as the standard for measuring bone mineral density (BMD). However, these techniques primarily assess structural changes and may fail to capture the underlying molecular alterations that precede visible bone loss. Consequently, there is increasing interest in advanced imaging modalities, such as [^18^F]-NaF PET, which can provide deeper insights into bone metabolism before noticeable structural changes occur [11,12].

[^18^F]-NaF-PET imaging quantifies bone turnover at a molecular level, and offers a new perspective on osteoporosis. This technique works by measuring the incorporation of [^18^F]-NaF into bone tissue, which reflects osteoblastic activity and mineralization processes. Notably, [^18^F]-NaF uptake is influenced by blood flow and the metabolic state of bone, allowing clinicians to observe changes in bone metabolism in real time [13]. Recent studies have demonstrated that [^18^F]-NaF-PET can differentiate between normal, osteopenic, and osteoporotic bone by analyzing plasma clearance and uptake values, indicating its potential for early diagnosis and monitoring the efficacy of pharmacotherapy [14,15].

As a molecular imaging tool, [^18^F]-NaF-PET is particularly beneficial for assessing the effects of various osteoporosis treatments. Research has shown that this technique can detect changes in bone metabolism before conventional markers or imaging methods reveal alterations. For example, it can capture differences in [^18^F]-NaF uptake in response to treatments such as bisphosphonates and anabolic agents. With its sensitivity to molecular changes in bone, [^18^F]-NaF-PET holds promise as a valuable tool for clinicians in tailoring treatment strategies, monitoring disease progression, and improving patient outcomes in those with metabolic bone diseases [16,17,18,19]. Further investigations into site-specific imaging may enhance understanding and application, paving the way for integrating [^18^F]-NaF-PET into routine clinical practice for osteoporosis management.

**Figure 1:**
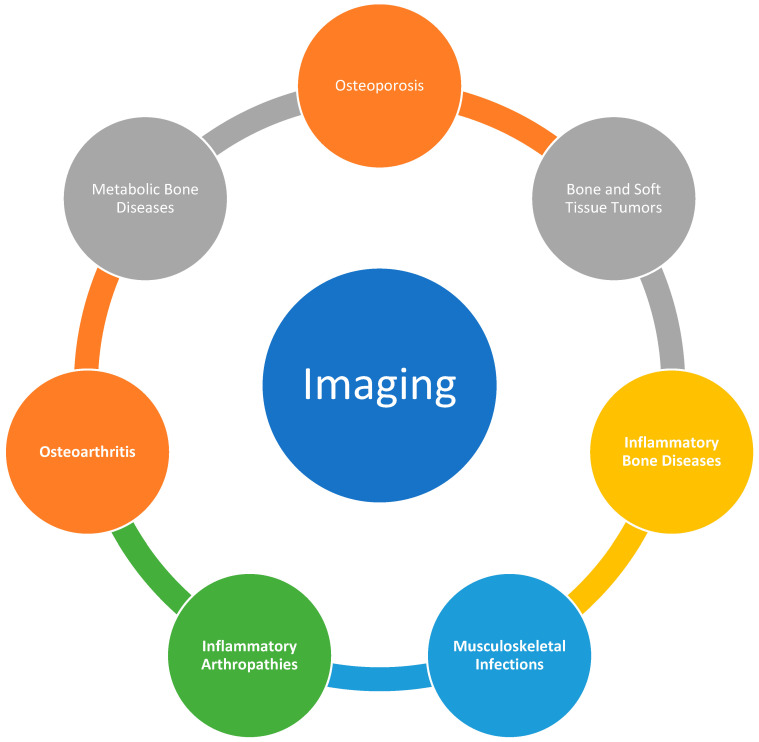
Musculoskeletal conditions where special radiological imaging (e.g., PET-CT) is used.

### 3.2. Bone Tumors and Soft Tissue Sarcoma

Soft tissue sarcomas (STS) are rare tumors that develop from mesenchymal tissue and account for less than 1% of all solid tumors in adults and about 7% of pediatric cancers. Although STS can occur at any age, they pose a significant health challenge, being responsible for 2% of all cancer-related deaths [20] Patients typically present with a palpable mass that enlarges over time, which may lead to symptoms caused by pressure on the surrounding nerves and blood vessels. The most common anatomical locations for STS are the extremities, with approximately 70% of cases arising in these areas. With advancements in imaging technology, particularly with PET-CT, there has been an increasing interest in utilizing [^18^F]-FDG PET imaging to enhance the diagnosis and management of STS [21].

While [^18^F]-FDG PET-CT is not usually the first imaging modality employed for the initial diagnosis of STS, it can play a crucial role in identifying malignant transformations in benign lesions. For instance, this imaging technique can detect the progression of a plexiform neurofibroma to a malignant peripheral nerve sheath tumor (MPNST). Although PET-CT cannot replace direct tissue biopsies, it can improve diagnostic outcomes by pinpointing hypermetabolic regions within heterogeneous lesions. Tumor grading is crucial for predicting biological behavior and clinical prognosis, and recent studies have shown that [^18^F]-FDG PET-CT scans aid in grading STS, correlating maximum standardized uptake values (SUVmax) with histological grades effectively [22,23].

Research has indicated that [^18^F]-FDG PET-CT is beneficial in distinguishing between benign and malignant STS. Various studies reported high sensitivity and specificity rates, particularly with an SUVmax threshold of 2.0 to 3.0, enabling the accurate differentiation of tumor grades [24]. The [^18^F]-FDG kinetics can further enhance the distinction between malignant and benign tumors; for instance, malignant tumors typically reach peak [^18^F]-FDG uptake much later than benign ones [25,26]. The technique has also outperformed traditional imaging modalities in assessing the stage of STS, particularly in detecting pulmonary and bone metastases, providing valuable information for current staging systems like the American Joint Committee on Cancer (AJCC) classification [27].

**Figure 2:**
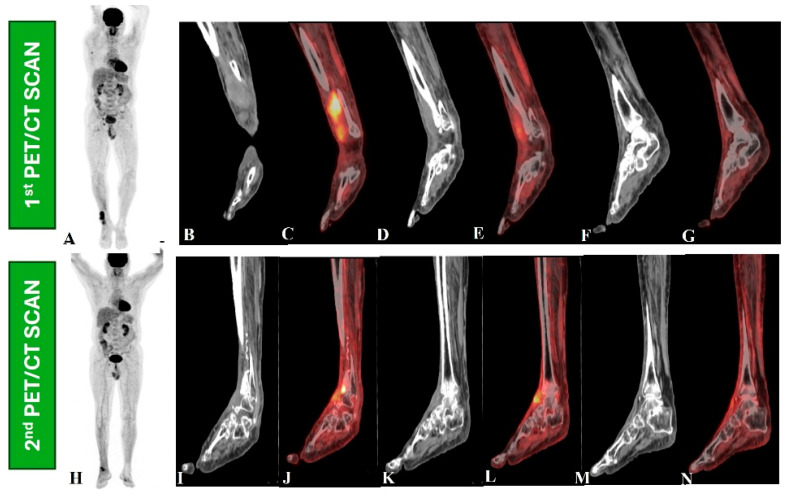
A 66-year-old male had wide-excision surgery for recurrent soft tissue sarcoma in the right leg. Following surgery, the CT component in the first PET/CT scan (upper row, **B**–**G**) revealed a mass lesion along the anterior aspect of the right distal tibia (**B**,**D**,**F**) and increased [^18^F]-FDG uptake on the maximum intensity projection (MIP) PET image (**A**) and fused PET and CT images (**C**,**F**), indicating active residual disease. The patient then received radiation therapy and returned for a response assessment PET/CT scan (lower row, **I**–**N**), which revealed ill-defined soft tissue thickening along the anterior aspect of the right distal tibia in a CT scan (**I**,**K**,**M**) and metabolically active cortical erosion with reduced [^18^F]-FDG avidity, suggesting a partial metabolic response (**H**,**J**,**L**,**N**).

**Figure 3:**
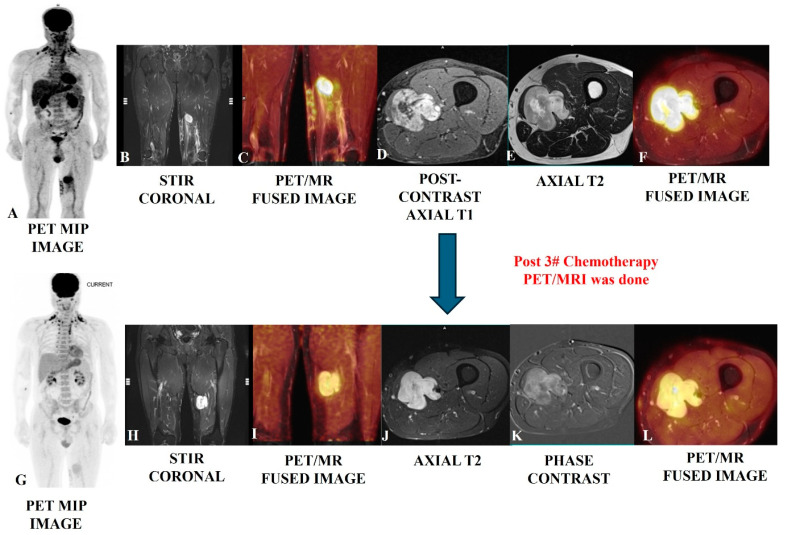
Post-operative case of myosarcoma of the left mid-thigh in a 45-year-old male evaluated using PET/MRI SCAN (upper row); the scan showed an altered-intensity heterogeneously enhanced bulky residual mass with lobulated, infiltrative margins in the tissue planes, which is well depicted in the MRI images (**B**,**D**,**E**), as well as increased [^18^F]-FDG metabolic avidity (**A**,**C**,**F**). A follow-up PET/MRI scan (bottom row) after three cycles of chemotherapy showed residual enhancing altered-intensity soft tissue mass with no gross change in bulk (**H**,**J**,**K**) and moderate regression in avidity (**G**,**I**,**L**), suggesting a partial response.

**Figure 4:**
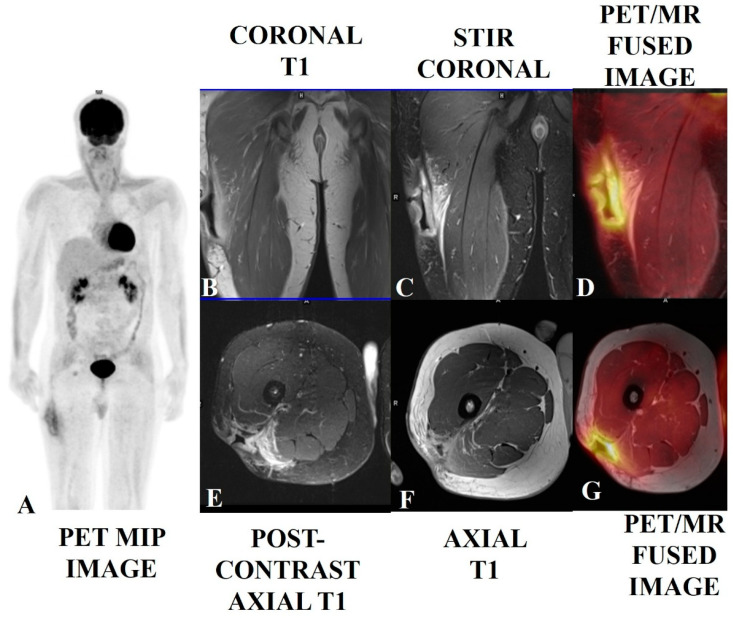
A soft tissue sarcoma (spindle cell tumor with mild atypia) in the right upper thigh of a 38-year-old male who had undergone local excision, with positive margins, followed by radiotherapy. The patient presented with a surgical scar with wound dehiscence and a deep excavated ulcer. The PET/MRI scan showed post-op changes with smooth diffusely enhanced thickening and no nodularity (**B**,**C**,**E**,**F**) and mild diffuse [^18^F]-FDG tracer uptake along the margins of the ulcerated lesion (**A**,**D**,**G**), suggesting more post-op/RT changes with a low probability of any underlying disease. This was proven via histology. Biopsies of enlarged and avid ilioinguinal and iliofemoral nodes were found to be reactive.

**Figure 5:**
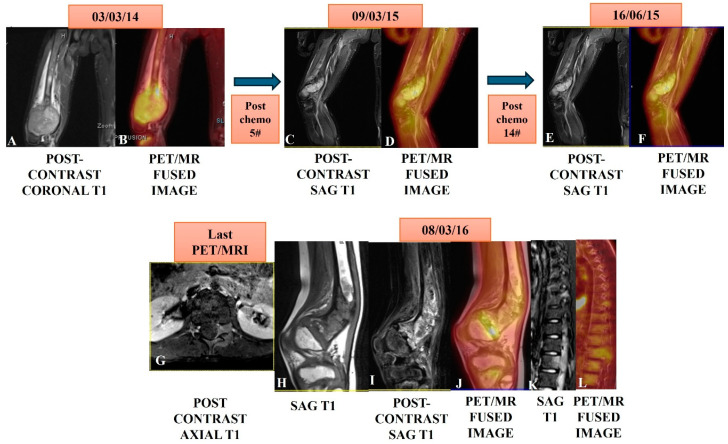
A case of osteosarcoma of the distal right femur in an eight-year-old male child showed an expansile enhancing and metabolically active mass in the distal femur (**A**,**B**) in a PET/MRI conducted on 03/03/14. The patient underwent serial PET/MRI studies after five cycles on 09/05/15 and then, after 14 cycles of chemotherapy on 16/06/15, showed marked regression in bulk and near complete metabolic regression in serial scans (**C**,**D**,**E**,**F**). A follow-up PET/MRI scan was taken after nine months on 08/03/16 and showed residual/recurrent enhancing (**H**,**I**) and a metabolically avid marrow lesion (**J**) in the meta-diaphysis of the distal femur. Note: the [^18^F]-FDG avid marrow lesion left posterior elements of D9 and L1 (**K**,**L**) are suggestive of metastasis with left spinal epidural soft issue extension at the L1 level (**G**).

Beyond diagnosis and staging, [^18^F]-FDG PET-CT is useful in evaluating treatment responses and monitoring for recurrence in STS patients [27,28,29]. It demonstrates superiority in distinguishing between scar tissue and active tumors, proving its effectiveness over traditional imaging methods [30,31,32]. The changes in metabolic activity are more reliable indicators of treatment response than size alterations; for example, a significant decrease in SUVmax after treatment correlates with better clinical outcomes [32,33]. Advancements in combined imaging methodologies, such as PET/MRI, are also showing promise, enhancing the detection and management of STS by integrating metabolic and anatomical data, thereby improving overall patient care [34,35,36]. Figure 2, Figure 3, Figure 4 and Figure 5 provide examples of some representative oncology cases of MSK imaging evaluated with PET (PET/CT or PET/MRI) for staging purposes and/or to assess treatment responses.

### 3.3. Inflammatory Bone Diseases

Positron emission tomography (PET), traditionally used in cancer diagnosis, is increasingly recognized for its value in inflammatory conditions. Specifically, Fluorodeoxyglucose ([^18^F]-FDG)-PET has proven effective in diagnosing and evaluating inflammation across various clinical areas. This is because the molecular and cellular processes underlying inflammation offer targets for molecular imaging [37,38].

The core principle behind [^18^F]-FDG uptake, the Warburg effect (increased glucose metabolism), is not exclusive to cancer. Activated inflammatory cells, such as neutrophils and macrophages, also exhibit heightened glycolysis. This “respiratory burst” leads to the rapid influx and metabolic trapping of [^18^F]-FDG, mirroring the process in malignant cells. For example, activated lymphocytes can dramatically increase [^18^F]-FDG uptake (up to 20-fold within 24 h) due to a shift to glycolysis [39].

In inflammatory bone diseases, [^18^F]-FDG-PET offers a non-invasive method to assess the disease’s extent and activity in vivo. Monitoring treatment responses in systemic inflammatory diseases, including bone conditions, poses significant challenges. The ability to rapidly and accurately evaluate therapeutic effectiveness is crucial for the timely discontinuation of ineffective treatments. [^18^F]-FDG-PET provides a valuable tool, enabling clinicians to visualize and quantify inflammatory activity [37,38,39].

### 3.4. Musculoskeletal Infections

Osteomyelitis remains a significant global health concern, necessitating prompt and accurate diagnosis for effective treatment and the prevention of complications. While MRI is highly sensitive in detecting early osteomyelitis, showing characteristic signal changes, [^18^F]-FDG-PET/CT has demonstrated superior diagnostic accuracy, particularly in chronic cases. Meta-analyses have shown [^18^F]-FDG-PET’s high sensitivity (96%) and specificity (91%) in detecting chronic osteomyelitis in axial and appendicular skeletons, outperforming other imaging modalities [40].

Hybrid [^18^F]-FDG-PET/MR imaging is emerging as a valuable tool for the diagnosis of musculoskeletal infections. In vertebral osteomyelitis and spondylodiscitis, PET/MR is preferred due to MRI’s superior soft tissue and bone marrow detail compared to CT. This combination allows for the precise assessment of soft tissue abscesses, surgical planning, and early detection, where [^18^F]-FDG-PET often surpasses MRI [41]. Studies suggest PET/MR offers comparable diagnostic utility to PET/CT in chronic osteomyelitis, aiding in accurate debridement planning by correlating [^18^F]-FDG uptake with MRI findings [42].

While other PET radiotracers such as ^11^C-methionine and [^68^Ga]-citrate have been explored, [^18^F]-FDG remains the most effective. [^124^I]-FIA (2′deoxy-2′-fluoro-B-D-arabinofuranosyl)-5-iodouracil), targeting microbial thymidine kinase, shows promise but requires further clinical validation [43].

[^18^F]-FDG-PET/CT is also valuable in assessing prosthetic joint infections (PJI) after arthroplasty, demonstrating high pooled sensitivity and specificity (87%) [37]. Notably, hip prosthesis infections show higher diagnostic accuracy than knee infections [38]. Beyond diagnosis, [^18^F]-FDG-PET/CT provides crucial information for surgical planning, including infection location and extent, periosteal reaction, osteolysis, joint stability, and soft tissue integrity, assisting orthopedic surgeons in optimal decision making [44,45,46].

### 3.5. Inflammatory Arthropathies

[^18^F]-FDG-PET effectively visualizes inflammatory activity in joints and surrounding tissues in various rheumatic diseases, demonstrating strong correlations with serological and clinical markers. For example, in rheumatoid arthritis (RA), [^18^F]-FDG uptake strongly correlates with clinically assessed joint inflammation, disease activity scores, and erythrocyte sedimentation rates. [^18^F]-FDG-PET aids in early RA detection and in the monitoring of treatment responses. Moreover, the distinct metabolic patterns observed in [^18^F]-FDG-PET may assist in differentiating between various inflammatory rheumatic conditions [47,48,49,50].

Beyond [^18^F]-FDG and [^18^F]-NaF, other PET tracers are being explored for RA imaging. [^68^Ga]-PRGD2, primarily highlighting angiogenesis in the synovium, shows lower muscle uptake than [^18^F]-FDG. [^11^C]-choline, like [^18^F]-FDG, correlates with synovial tissue volume on MRI but offers faster joint visualization (10 min). While [^11^C]-PK11195 and [^11^CD]-deprenyl have been studied, the development of tracers targeting specific molecular components of synovitis holds promise for a deeper understanding of the disease’s molecular basis [51,52].

[^18^F]-FDG-PET/MR imaging is also gaining traction in RA imaging, offering the advantage of simultaneously visualizing marrow oedema and synovitis. Studies have demonstrated its feasibility for hand imaging in early RA, though further research is needed to evaluate its potential fully [53].

### 3.6. Osteoarthritis

While radiography remains the primary diagnostic tool for OA, its limitations in 3D visualization and soft tissue evaluation have led to the increasing use of MRI, which provides a detailed assessment of various joint structures. PET imaging, using [^18^F]-FDG and [^18^F]-NaF, offers valuable insights into the metabolic changes associated with OA, revealing inflammation, infection, and bone remodeling [54]. Increased [^18^F]-FDG uptake in knee OA correlates with MRI-detected bone marrow lesions (BMLs) and rising [^18^F]-FDG uptake in the joint space, indicative of secondary inflammation and cartilage deterioration. [^18^F]-fluoride PET shows higher uptake in progressive OA and acute pain, even in the early stages without radiographic joint space narrowing. [^18^F]-NaF highlights subchondral bone alterations, including increased blood flow and remodeling, preceding and following the onset of OA, correlating with pain and later cartilage changes [55].

The combination of PET and MRI is proving particularly beneficial in OA research. Studies have shown that co-registered [^18^F]-NaF PET with MRI can quantitatively assess metabolic changes in both osseous and non-osseous structures in early OA [55]. Human studies have demonstrated the simultaneous interplay of bone remodeling (PET), cartilage biochemistry, and quantitative MR biometrics in knee OA. Notably, PET/MRI can detect metabolic abnormalities in the subchondral bone that appear normal on MRI, suggesting early functional changes preceding structural damage. BMLs on MRI show high [^18^F]-NaF uptake but minimal [^18^F]-FDG uptake, indicating bone remodeling rather than primary inflammation [56].

Further research using [^18^F]-NaF PET/MRI has revealed correlations between increased cartilage T2 times, subchondral bone [^18^F]-NaF SUVmax, and ACL reconstruction, potentially serving as early OA indicators [57]. Studies have also shown correlations between quantitative MR of the hip and femur and PET evidence of bone remodeling, as well as between abnormal bone metabolism and increased bone perfusion in regions with osteophytes, cartilage lesions, and BMLs [58,59]. Additionally, associations have been observed between [^18^F]-NaF SUVmax and dynamic CEMRI K^trans^ values, synovitis severity, and subchondral bone [^18^F]-NaF uptake. Kinetic features of [^18^F]-NaF uptake in the subchondral bone can quantify bone physiology’s role in OA onset and progression [60,61].

**Figure 6:**
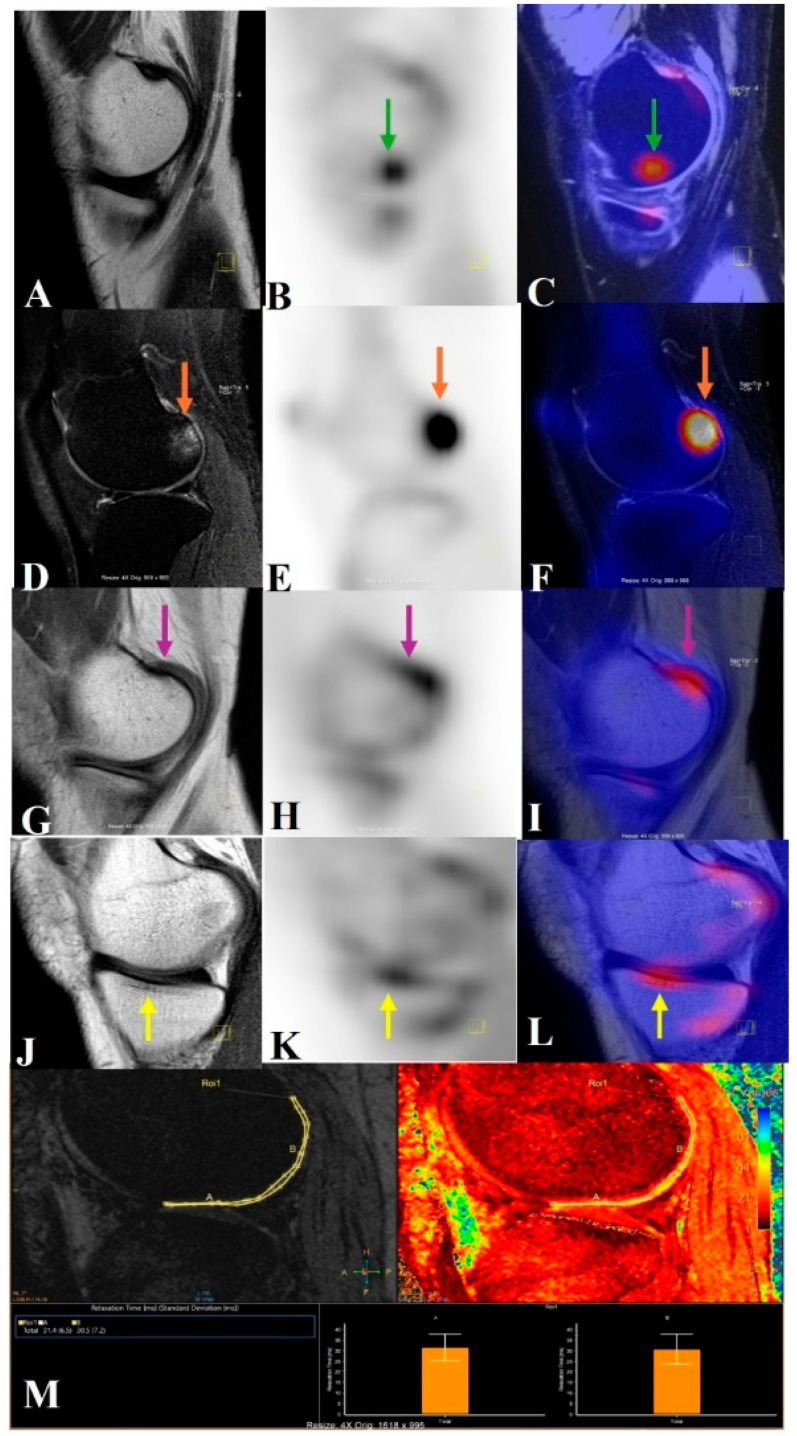
This image illustrates how knees with osteoarthritis can be seen holistically using PET/MRI. A PET/MRI scan of the knee shows high tracer uptake in the medial central femur region (**B**,**C**) without any morphological changes (green arrow) (subchondral magic spots) in the corresponding sagittal T2 SPIR (spectral presaturation with inversion recovery) image (**A**). High signal intensity changes, i.e., grade 2 BML (orange arrow) in the lateral posterior femur on a T2 SPIR image (**D**), with high tracer uptake on the sagittal PET image (**E**) also spatially correlated in the fused PET/MRI image (**F**). Osteophyte in the medial posterior femur (pink arrow) in the T1 TSE image (**G**), with high tracer uptake in the sagittal PET image (**H**) also spatially correlated in the fused PET/MRI image (**I**). Sclerotic changes in the medial central tibial region (yellow arrow) in the T1 TSE image (**J**), with high tracer uptake in the sagittal PET image (**K**) also spatially correlated with the fused PET/MRI image (**L**). The lateral central and posterior femur cartilage had longer relaxation times, according to the T2* relaxometry cartogram superimposed on the sagittal T2 SPIR picture (**M**).

The analysis conducted by Jena et al. [^18^F]-NaF PET/MRI revealed compartment-specific differences in osteophyte and BML SUVmax, highlighting differential bone remodeling [62]. They also found increased [^18^F]-NaF uptake in BMLs and osteophytes correlating with higher MRI osteoarthritis knee score (MOAKS) grades and associated cartilage degradation. Discordance between structural MRI findings and [^18^F]-NaF uptake at cruciate ligament insertion points was observed. Subchondral SUVmax significantly correlated with T2* relaxometry and cartilage grades, with BMLs showing the highest metabolic activity [63]. These studies underscore the potential of PET/MRI to provide valuable insights into OA pathophysiology, aiding in early detection, disease monitoring, and the development of targeted therapies. Figure 6 demonstrates various changes in PET-MRI of the knee in a case of OA.

### 3.7. Metabolic Bone Diseases

#### 3.7.1. Paget’s Disease

Paget’s disease (PD) is a chronic bone disorder characterized by abnormal bone remodeling, primarily affecting the axial skeleton. While [^18^F]-FDG PET often shows minimal uptake in benign PD, this characteristic can help differentiate it from Paget’s sarcoma [64]. Case studies highlight the utility of PET in diagnosing PD, particularly when distinguishing it from other bone conditions such as metastasis or osteomyelitis. For instance, subtle [^18^F]-FDG uptake combined with vigorous osteoblastic activity on bone scans or high [^18^F]-NaF uptake correlating with radiographic findings and biochemical markers, such as elevated alkaline phosphatase, aids in accurate diagnosis [65,66].

Furthermore, [^18^F]-NaF PET/CT proves valuable in confirming PD, especially when radiographic findings suggest other possibilities such as metastatic lesions [67]. Dynamic [^18^F]-NaF PET also plays a crucial role in monitoring treatment responses, with SUVmax fluctuations mirroring kinetic indices, simplifying clinical applications, and eliminating the need for complex dynamic acquisition and arterial blood sampling [68]. These findings underscore the importance of PET, mainly [^18^F]-NaF PET/CT, as a supplementary diagnostic tool and a means of monitoring treatment efficacy in Paget’s disease.

#### 3.7.2. Osteomalacia

Oncogenic osteomalacia, a rare paraneoplastic syndrome, is caused by benign mesenchymal tumors that can be challenging to locate using traditional imaging. While [^18^F]-FDG PET/CT can detect these tumors due to their relatively high [^18^F]-FDG uptake, its sensitivity is limited [69,70,71,72]. Case studies highlight the difficulty of identifying these tumors using radiography and physical examinations, emphasizing the advantages of PET/CT, such as whole-body imaging and the visualization of the tumor itself rather than reactive bone changes [73].

Recent studies have demonstrated the superiority of [^68^Ga]-DOTATATE PET/CT over [^18^F]-FDG PET/CT and other conventional imaging modalities in detecting tumors causing oncogenic osteomalacia. This is particularly crucial for preventing treatment delays in this debilitating condition [74,75,76]. Case reports illustrate how [^68^Ga]-DOTATATE PET/CT successfully identified tumors missed by other imaging techniques, correlating with elevated FGF-23 levels and somatostatin receptor overexpression. The effectiveness of [^68^Ga]-DOTATATE PET/CT in localizing phosphaturic mesenchymal tumors in tumor-induced osteomalacia (TIO) patients has been consistently observed, with clinical symptom resolution following surgical excision [77].

Prospective investigations have further validated the efficacy of [^68^Ga]-DOTATATE PET/CT, revealing significantly higher detection rates compared to [^18^F]-FDG PET/CT and [^99m^Tc]-HYNIC-TOC scintigraphy. Studies show a strong correlation between SUVmax, tumor size, and somatostatin receptor 2 (SSTR2) expression [78]. [^68^Ga]-DOTATOC PET/CT has also proven effective in identifying culprit tumors missed by conventional radiological and nuclear medicine imaging, supporting precise diagnosis, and guiding surgical intervention in TIO [79]. These findings highlight the pivotal role of [^68^Ga]-DOTATATE PET/CT in the diagnostic workup of oncogenic osteomalacia.

Table 2 summarizes the mechanisms, key applications, advantages and limitations of the various imaging techniques used in a number of MSK conditions.

## 4. Limitations

While this review provides valuable insights into musculoskeletal disorders (MSDs) and the potential of advanced imaging techniques such as PET, it has limitations. We acknowledge that the majority of the images of the case studies used in this review are of malignant pathology, because, in our center, this modality is mostly used for cancer patients; hence, we were unable to provide suitable images of benign tumors in this review. The other limitations include the relatively narrow scope of the available literature on PET applications in MSDs, which may affect the comprehensiveness of the analysis. Furthermore, the review primarily focuses on radiotracers such as [^18^F]-FDG and [^18^F]-NaF while overlooking other emerging radiotracers that could also be significant in MSD diagnosis and management. Additionally, the review may not account for variations in imaging protocols and the technical challenges associated with implementing PET imaging in clinical settings, which could influence its accessibility and practicality. Lastly, while the discussion centers on integrating advanced imaging modalities, it may not sufficiently address the potential economic implications or the need for interdisciplinary collaboration to translate these innovations into routine clinical practice.

## 5. Future Directions

Future directions in MSK imaging should focus on enhancing the integration of advanced imaging modalities, such as PET, with established techniques like MRI and CT to provide a more comprehensive understanding of MSD [2,3]. Research should prioritize the development of novel radiotracers that target specific molecular pathways and inflammatory markers associated with various MSK conditions, enabling more accurate and personalized diagnoses [80]. Furthermore, there is a need for large-scale clinical trials to validate the efficacy and safety of PET imaging in diverse patient populations and various drug trials [81,82,83]. Collaboration between researchers, clinicians, and technologists is essential to advancing imaging protocols and standardizing methodologies for better reproducibility. Additionally, incorporating artificial intelligence (AI) and machine learning (ML) into image analysis could improve diagnostic accuracy and facilitate the early detection of MSDs. Ultimately, these efforts aim to create a more holistic approach to managing MSDs, leading to improved patient outcomes and enhanced quality of life [84,85].

## 6. Conclusions

This review highlights the significant burden that MSDs impose on public health and underscores the limitations of current diagnostic methods, particularly conventional imaging techniques. The exploration of advanced imaging modalities, specifically PET, emerges as a promising avenue for enhancing the early diagnosis and monitoring of MSDs. The unique capabilities of PET, especially in assessing metabolic and molecular activity, offer valuable insights that traditional imaging may overlook. Furthermore, the review demonstrates the potential of using established radiotracers like [^18^F]-FDG and [^18^F]-NaF to improve our understanding of inflammatory processes and bone metabolism in various conditions. Recommendations for future research emphasize the importance of developing novel radiotracers and integrating advanced imaging techniques to bridge existing knowledge gaps. Advancing these strategies will be crucial for improving diagnostic accuracy, tailoring treatment plans, and ultimately enhancing patient outcomes in musculoskeletal health.

## Figures and Tables

**Table 1:** Summary of the radiotracers for musculoskeletal imaging. Adapted from Kogan et al. [2] and Gholamrezanezhad et al. [3].

Radiotracer	Target/ Mechanism	Primary Applications in Musculoskeletal Imaging	Advantages	Limitations
[^18^F]-FDG (Fluorodeoxyglucose) [2]	Glucose uptake, metabolic activity	- Inflammation (arthritis, infection) - Identifying areas of increased metabolic demand	- Effective for detecting inflammatory processes - Widely available and commonly used	- Less effective in purely bone-related metabolic changes - Can produce false positives due to non-specific glucose uptake (e.g., muscle activity)
[^18^F]-NaF (Sodium Fluoride) [3]	Bone metabolism, hydroxyapatite exchange	- Bone metastases (especially from prostate and breast cancers) - Bone turnover and repair (fracture healing, arthritis, metabolic bone disorders) - Newly mineralizing bone	- Highly sensitive to bone metabolic activity - Excellent for evaluating bone turnover - Useful when FDG is less sensitive to bone-related issues.	- Primarily targets bone; less useful for soft tissue inflammation - Can be affected by renal function
[^68^Ga]-PSMA-11 [7]	Binding to PSMA	Metastasis in prostate cancer	- More precise detection of prostate cancer than current standard techniques - Can detect tumours in the prostate, pelvis, and body - Can help identify tumours that have migrated	- Non-specific uptake in neoplastic and inflammatory diseases.
[^11^C]-Choline [8]	Cell proliferation	Proliferative changes in arthritic synovium	- It is a precursor for the biosynthesis of phosphatidylcholine, which is an essential component of the cell membrane	Short half-life
[^11^C]-(R)-PK11195 [9]	Activated Macrophages	Specificity to active inflammation	- [^11^C]-PK11195 binds to activated macrophages in the vessel wall. - It’s a prototypical TSPO radiotracer.	Short Half-life
[^18^F]-FTC-146 [10]	Binding to Sigma 1 Receptor	Active neuropathic pain	- High S1R selectivity, allowing for the targeted visualization of S1R activity	- Metabolic limitations in certain species - Complex synthesis - Limited clinical translation

(PSMA—prostate-specific membrane antigen; TSPO—translocator protein; S1R—Sigma 1 receptor; [18F] FTC—Fluorine-18 tracer compound).

**Table 2:** Mechanism, key applications, advantages and limitations of various imaging techniques in musculoskeletal conditions.

Imaging Technique	Condition	Target/Mechanism	Key Applications	Advantages	Limitations
[^18^F]-NaF PET [16,17,18,19]	Osteoporosis	Bone turnover, osteoblastic activity	- Early detection of bone metabolic changes - Monitoring treatment efficacy - Differentiating bone states (normal, osteopenic, osteoporotic)	- Quantifies bone turnover at a molecular level - Detects changes before structural alterations - Sensitive to treatment responses	- Less widely available than DXA - Higher cost - Requires specialized equipment and expertise
[^18^F]-NaF PET [60,61,62]	Osteoarthritis (OA)	Bone remodelling, subchondral bone changes	- Detecting early metabolic changes - Assessing bone turnover and remodelling - Correlating with pain and cartilage changes	- Highlights subchondral bone alterations - Detects changes before structural damage	Primarily targets bone
[^18^F]-NaF PET/CT [64,65,66]	Paget’s Disease (PD)	Bone turnover, osteoblastic activity	- Confirming diagnosis - Monitoring treatment response	- Provides metabolic and anatomical information - Dynamic PET simplifies clinical application	Requires specialized equipment and expertise
[^18^F]-NaF PET/MR [60,61,62]	Osteoarthritis (OA)	Bone remodeling, cartilage biochemistry, MR biometrics	- Simultaneous assessment of bone and soft tissue changes - Detecting early functional changes	- Combines metabolic and anatomical information - Detects abnormalities not visible on MRI alone	Requires specialized equipment and expertise
[^18^F]-FDG PET [57,58,59]	Osteoarthritis (OA)	Glucose uptake, inflammation	- Detecting inflammation and secondary inflammation - Correlating with MRI-detected bone marrow lesions	Provides metabolic information	Minimal uptake in bone remodelling
[^18^F]-FDG PET [37,38,39]	Inflammatory Bone Diseases	Glucose uptake, inflammatory cell activity	- Assessing disease extent and activity - Monitoring treatment response	- Non-invasive - Visualizes and quantifies inflammatory activity	Not specific to bone inflammation, can show inflammation anywhere.
[^18^F]-FDG PET [64,65,66]	Paget’s Disease (PD)	Glucose uptake, metabolic activity	- Differentiating benign disease from Paget’s sarcoma	- Aids in differential diagnosis	Often shows minimal uptake in benign PD
[^18^F]-FDG PET [51,52]	Inflammatory Arthropathies	Glucose uptake, inflammatory activity	- Visualizing joint inflammation - Early RA detection - Monitoring treatment response - Differentiating rheumatic conditions	- Correlates with clinical and serological markers - Detects early changes	Not specific to RA, can show inflammation anywhere.
[^18^F]-FDG PET/CT [21,22,23,24,25,26,27,28]	Bone Tumors and Soft Tissue Sarcoma (STS)	Glucose uptake, metabolic activity	- Identifying malignant transformations - Tumor grading (SUVmax correlation) - Staging (detecting metastases) - Evaluating treatment response - Monitoring recurrence	- High sensitivity and specificity for malignancy - Distinguishes scar tissue from active tumours - Provides metabolic information beyond structural changes	- Not a primary diagnostic tool for initial STS diagnosis - Requires tissue biopsy for definitive diagnosis - Can have false positives due to inflammation
[^18^F]-FDG PET/CT [69,70,71,72,73]	Osteomalacia	Glucose uptake, metabolic activity	- Detecting tumours causing osteomalacia	Whole-body imaging	Limited sensitivity
[^18^F]-FDG PET/CT [40]	Musculoskeletal Infections	Glucose uptake, inflammatory cell activity	- Diagnosing chronic osteomyelitis - Assessing prosthetic joint infections - Surgical planning	- High sensitivity and specificity - Superior to MRI in chronic cases - Provides information for surgical planning	Can have false positives due to non-infectious inflammation
	Musculoskeletal Infections	Glucose uptake, soft tissue and bone marrow detail	- Vertebral osteomyelitis/spondylodiscitis - Precise assessment of soft tissue abscesses	- Combines metabolic and anatomical information - Superior soft tissue detail compared to PET/CT	Less widely available than PET/CT
[^18^F]-FDG PET/MR [41]	Inflammatory Arthropathies	Glucose uptake, marrow oedema, synovitis	- Simultaneous visualization of marrow oedema and synovitis - Hand imaging in early Rheumatoid Arthritis	- Combines metabolic and anatomical information	Further research needed to fully evaluate the potential
[^68^Ga]-DOTA-TATE PET/CT [74,75,76,77,78,79]	Osteomalacia	Somatostatin receptor binding	- Detecting tumors causing osteomalacia - Localizing phosphaturic mesenchymal tumors	- Superior to [^18^F]-FDG PET/CT - High detection rates	Requires specialized radiotracers and expertise
MRI [55,56,57,58,59]	Osteoarthritis (OA)	Soft tissue and cartilage assessment	- Detailed assessment of joint structures - Detection of bone marrow lesions	- High soft tissue resolution - Provides structural information	Less sensitive to early metabolic changes
MRI [34,35,36]	Bone Tumors and Soft Tissue Sarcoma (STS)	Soft tissue detail, anatomical information	Initial diagnosis, surgical planning	High soft tissue resolution	Less sensitive to metabolic activity.
MRI [42]	Musculoskeletal Infections	Signal changes in bone marrow and soft tissue.	Early detection of osteomyelitis	High sensitivity for early disease.	[^18^F]-FDG-PET/CT has superior diagnostic accuracy in chronic cases.
Radiography [54]	Osteoarthritis (OA)	Structural changes (joint space narrowing, osteophytes)	Primary diagnostic tool	- Widely available - Established clinical guidelines	- Limited 3D visualization - Poor soft tissue evaluation
DXA (Dual-Energy X-ray Absorptiometry) [11,12]	Osteoporosis	Bone mineral density (BMD)	- Standard for diagnosing osteoporosis - Assessing fracture risk	- Widely available - Relatively low radiation dose - Established clinical guidelines	- Primarily assesses structural changes - May not capture early molecular changes - Limited information on bone quality
Radiography [54]	Osteoarthritis (OA)	Structural changes (joint space narrowing, osteophytes)	- Primary diagnostic tool	- Widely available - Established clinical guidelines	- Limited 3D visualization - Poor soft tissue evaluation
DXA (Dual-Energy X-ray Absorptiometry) [11,12]	Osteoporosis	Bone mineral density (BMD)	- Standard for diagnosing osteoporosis - Assesses fracture risk	- Widely available - Relatively low radiation dose - Established clinical guidelines	- Primarily assesses structural changes - May not capture early molecular changes - Limited information on bone quality

## Data Availability

The data used in this research is publicly available.
